# Association between plasma trans fatty acid levels and rheumatoid arthritis: a cross-sectional study using NHANES 1999–2000 and 2009–2010 data in US adults

**DOI:** 10.3389/fnut.2024.1413091

**Published:** 2024-12-12

**Authors:** Tanjian Li, Na Jiang, Xin Liang, Xinya Li, Yaqin Li, Yuting Huang, Yu Wang

**Affiliations:** ^1^School of Nursing, Jinan University, Guangzhou, China; ^2^School of Health, Binzhou Polytechnic, Binzhou, China; ^3^School of Nursing, Jinan University, The First Affiliated Hospital of Jinan University, The Community Health Service Center of Jinan University, Guangzhou, China

**Keywords:** NHANES, trans fatty acids, linolelaidic acid, rheumatoid arthritis, cross-sectional study

## Abstract

**Objective:**

While earlier research has indicated that trans fatty acids (TFAs) are detrimental to cardiovascular health as well as other conditions, the purpose of this study is to look into any possible connections between trans fatty acids and rheumatoid arthritis (RA).

**Methods:**

The NHANES database provided the data for this study, covering two periods: 1999–2000 and 2009–2010. The correlation between plasma TFAs (linolelaidic acid, vaccenic acid, palmitelaidic acid, and elaidic acid) and RA was examined using weighted univariate and multivariate regression analyses as well as analysis of subgroups. Additionally, this study used restricted cubic spline curves to investigate the non-linear relationship between them.

**Results:**

This study included 2,938 patients, of whom 222 (7.56%) had RA. Multivariate logistic regression analysis showed that levels of linolelaidic acid were linked to a higher risk of RA (odds ratio = 1.39, 95% confidence interval = 1.05–1.85, *p* = 0.025) after accounting for all other variables. No significant effect on this association was found in interaction tests. A linear association between linolelaidic acid and RA was demonstrated in the limited cubic spline regression model. For RA, linolelaidic acid exhibited a critical value of 0.98.

**Conclusion:**

Findings suggesting a possible link between elevated plasma TFA levels and an increased risk of RA offer fresh perspectives on RA prevention through dietary interventions.

## Introduction

Rheumatoid arthritis (RA) is an autoimmune disease that causes pain, inflammation, and stiffness in the synovial joints (1). It is crucial to understand the profound burden of this disease, as it affects not only the patient’s health but also their mobility and overall quality of life (2). The Global Burden of Disease survey indicated that approximately 18 million individuals, or approximately 0.6% of adults worldwide, suffer from rheumatoid arthritis, making it a global public health concern (3). The number of people with RA is expected to increase, reaching 31.7 million worldwide by 2050, according to projections from the disease (4). As the population ages, RA poses a significant public health challenge, and early detection and intervention are critical. Dietary variables are increasingly thought to have a significant influence on the development of RA, even if the actual origin of the condition is still unclear.

There are two primary sources of trans fatty acids (TFAs) in the human diet: “industrial” and “natural.” Fish or vegetable oils that are liquid and contain unsaturated fatty acids can be partly hydrogenated during manufacture to create artificial or industrial trans fats. In contrast, microorganisms in the rumen of ruminants, including cattle, sheep, and goats, make natural trans fats by converting fatty acids from feed (5, 6). It is worth noting that ruminant foods are generally low in natural trans fats (usually 2–9%), and industrial trans fats are the largest source of dietary trans fats in humans. Frequently discovered in processed foods found in grocery stores, industrial trans fats are utilized in commercially baked goods (such as chocolate, cakes, and biscuits), margarine, baking shortening, frozen and fried foods (such as pies), and packaged snacks (such as potato chips; (6)). Research has demonstrated that exposure to large amounts of industrial trans fats can have negative impacts on human health, these include cardiovascular disease, cancer, obesity, insulin resistance, type 2 diabetes, and illnesses of the reproductive system (7–9).

TFAs have been linked to inflammation, which is a major contributing factor in RA. Research has indicated that the consumption of trans fats raises the blood levels of inflammatory indicators such as C reactive protein (CRP), interleukin-1β (IL-1β), tumor necrosis factor-α (TNF-α), chemokine C-C gamma-ligand 2 (CCL2), and interleukin-6 (IL-6) (10, 11). Elevated levels of these markers have also been linked to RA (12, 13). Recent research has investigated the link between diet and RA, with some studies suggesting that a diet high in inflammatory potential can increase the risk of developing RA (14).

Estimating levels of TFA intake is typically conducted through dietary questionnaires; however, their accuracy may be compromised by residual confounding variables such as misclassification, incomplete assessment, or unmeasured components (15). Nonetheless, plasma TFAs may represent food consumption throughout the previous 6 to 12 weeks and can be used as a more accurate biomarker for measuring TFA intake (16). This method can establish a stronger relationship with adverse outcomes (17), as plasma TFA levels are also associated with dietary inflammatory markers (18).

Considering the link between trans fats and inflammation, it is reasonable to assume that these fats are associated with RA, although further research is needed to explore this relationship in more detail.

The National Health and Nutrition Examination Survey (NHANES) is a survey designed to gather data regarding the nutritional and health conditions of the US population. To cover the whole US population, the survey uses a multistage, hierarchical, probabilistic cluster sampling design (19). Nevertheless, no studies have examined the connection between plasma TFAs and RA using the NHANES database as of yet. This research seeks to close this knowledge gap by examining the connection between TFAs and RA in NHANES participants. Plasma TFA levels are thought to be greater in RA patients.

## Materials and methods

### Survey statement

The NHANES, a thorough census of the US population, is carried out by the Centers for Disease Control and Prevention’s National Center for Health Statistics (NCHS, CDC). It collects data on various aspects such as demographics, health and disease, socioeconomic status, lifestyle, diet, and laboratory test samples. The report is made available to the public every 2 years. To ensure that the coverage is representative, the NHANES selects a nationally representative sample that is dispersed around the nation using a layered multiple phases method based on random sampling. To ensure informed consent, each subject selected for the survey signs a written consent form. The NHANES has been authorized by the National Center for Health Statistics Ethics Review Board. The public can access the NHANES datasets, documentation, and agreements via www.cdc.gov/nchs/nhanes/.

### Population

Data were collected for two 2-year cycles, specifically during 1999–2000 and 2009–2010, as both plasma TFA concentrations and RA data were available during these cycles (20). Standardized processes and data collection methods were adhered to by conducting all measurements and testing in transportable test facilities that were set up on the premises.

In this investigation, particular exclusion criteria were used to guarantee the truthfulness and accuracy of the results. The exclusion criteria for this study were based on the following: (1) individuals under the age of 20, as we are primarily concerned with adults; (2) women who are expecting, as pregnancy may alter their metabolic profile; (3) those lacking data on RA, as their incorporation might induce distortion into the analysis of this research; (4) participants who lacked plasma TFA data, as these variables are essential for determining the correlation between TFAs and RA; and (5) individuals without covariate data.

### Measurement of plasma TFAs

**Figure 1:**
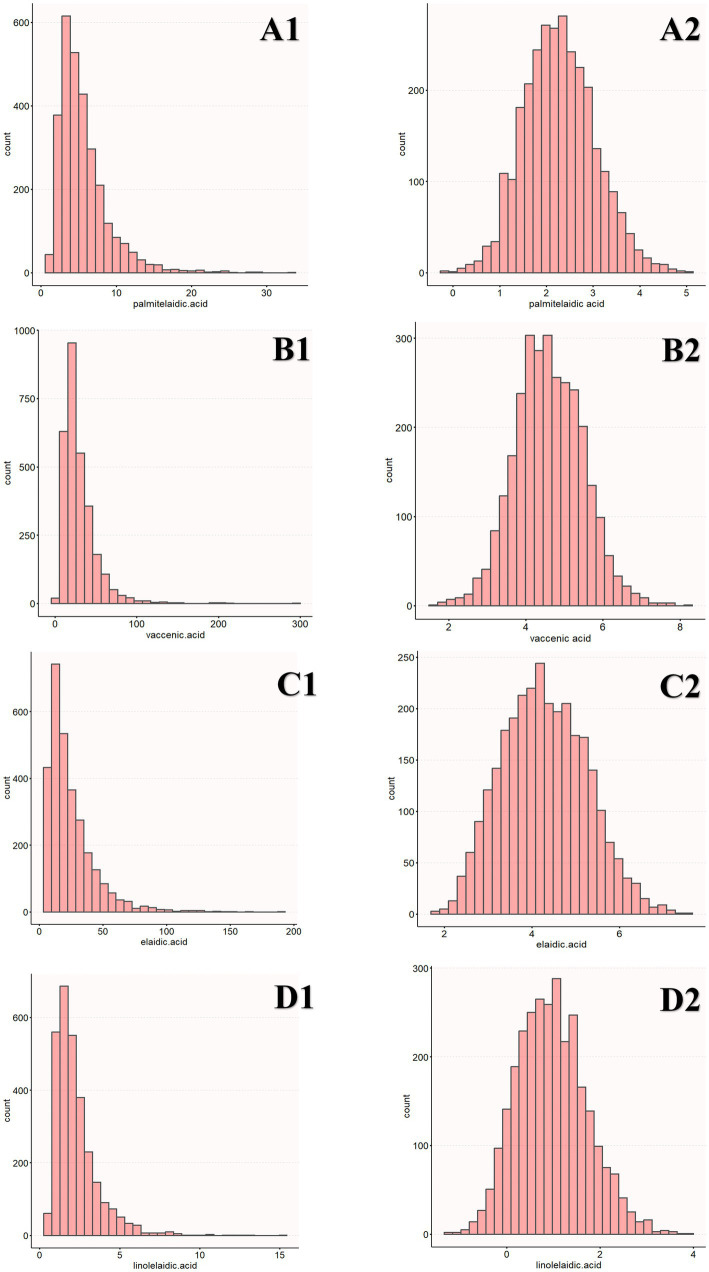
Distribution of TFAs **(A1, B1, C1, D1)**. The distribution of log2-transformed TFAs **(A2, B2, C2, D2)**.

Before eating, the participants’ blood samples were collected from their veins in the morning, and their serum was frozen and stored at −80°C. GC/MS was used to determine the levels of four types of TFAs (linolelaidic acid, elaidic acid, vaccenic acid, and palmitelaidic acid) present in the plasma. Lagerstedt et al. described the measurement technique, which was carried out in accordance with an extensive data quality assurance procedure for analytical quality control (21). The four types of TFAs included in this study are representative of the blood (22). For instance, the predominant isomer of TFAs found in industrial sources is ellagic acid. The predominant TFA isomer in bovine fat is the trans-isomer of vaccenic acid, which is a precursor to conjugated linoleic acid. Palmitoleic acid is mostly found in ruminant meat and milk, whereas linolelaidic acid is partly generated from hydrogenated oils. Additionally, the plasma trans fatty acids were transformed by log2 before regression analysis was performed since the distribution was right-skewed (Figure 1).

### Evaluation of RA

Specifically, the self-report questionnaires (MCQ160a), (MCQ191), and (MCQ190) were used to diagnose arthritis. Participants were given the choice to select “yes” or “no” when asked if they had ever been informed that they had arthritis by a physician or other healthcare provider. Participants were asked to identify the type of arthritis they had to be evaluated for rheumatoid arthritis. Response possibilities were psoriatic arthritis, osteoarthritis, rheumatoid arthritis, other, rejected, and do not know.

### Evaluate covariates of interest

This study has included some covariates that might have an impact on RA. Age is one of the important factors, and it is measured as a continuous variable. Furthermore, the participants were categorized into two age groups: those aged 50 years or under, and those over 50 years. Other covariates that were included in the study are gender, ethnicity (Mexican American, non-Hispanic black, non-Hispanic white, and other), education level (less than high school, completed high school, above high school), poverty-to-income ratio (PIR; <1.3, 1.3–3.5, ≥3.5), marital status (married or living with partners, widowed, divorced, separated, or never married), body mass index (BMI; <25, 25–30, and >30 kg/m^2^), work activity (yes, no), smoking status (never, former, now), alcohol consumption (yes, no), hypertension (yes, no), and diabetes (yes, no).

### Statistical analysis

The Free Statistics analysis platform (Version 1.9.2, Beijing, China) and R Statistical Software (Version 4.3.2, The R Foundation) were used for all analyses. The study used a significance level of *p* < 0.05 and excluded participants who lacked covariate data. Continuous variables were expressed as mean ± standard deviation (SD) or median and interquartile distance (IQR), and the *t*-test or Mann–Whitney U-test was used for inter-group comparisons. Categorical variables were compared using frequency (%) and the *χ*^2^ tests. Since TFAs are a skewed distribution, log2 transformation was performed on TFAs in regression analysis. The study tested the relationship between TFAs and RA in three models using a weighted bidirectional logistic regression model. There was not an adjustment variable in Model 1. Model 2 took PIR, education level, age, gender, marital status, and ethnicity into account. Model 3 included adjustments for work activity, BMI, smoking status, alcohol consumption, diabetes, and hypertension. The quartile was applied to plasma TFAs linked to RA as a categorical variable. The research also included cross-tabulations and additional stratification by ethnicity, age, gender, PIR, BMI, work activity, diabetes, hypertension, smoking status, and alcohol consumption.

## Results

**Figure 2:**
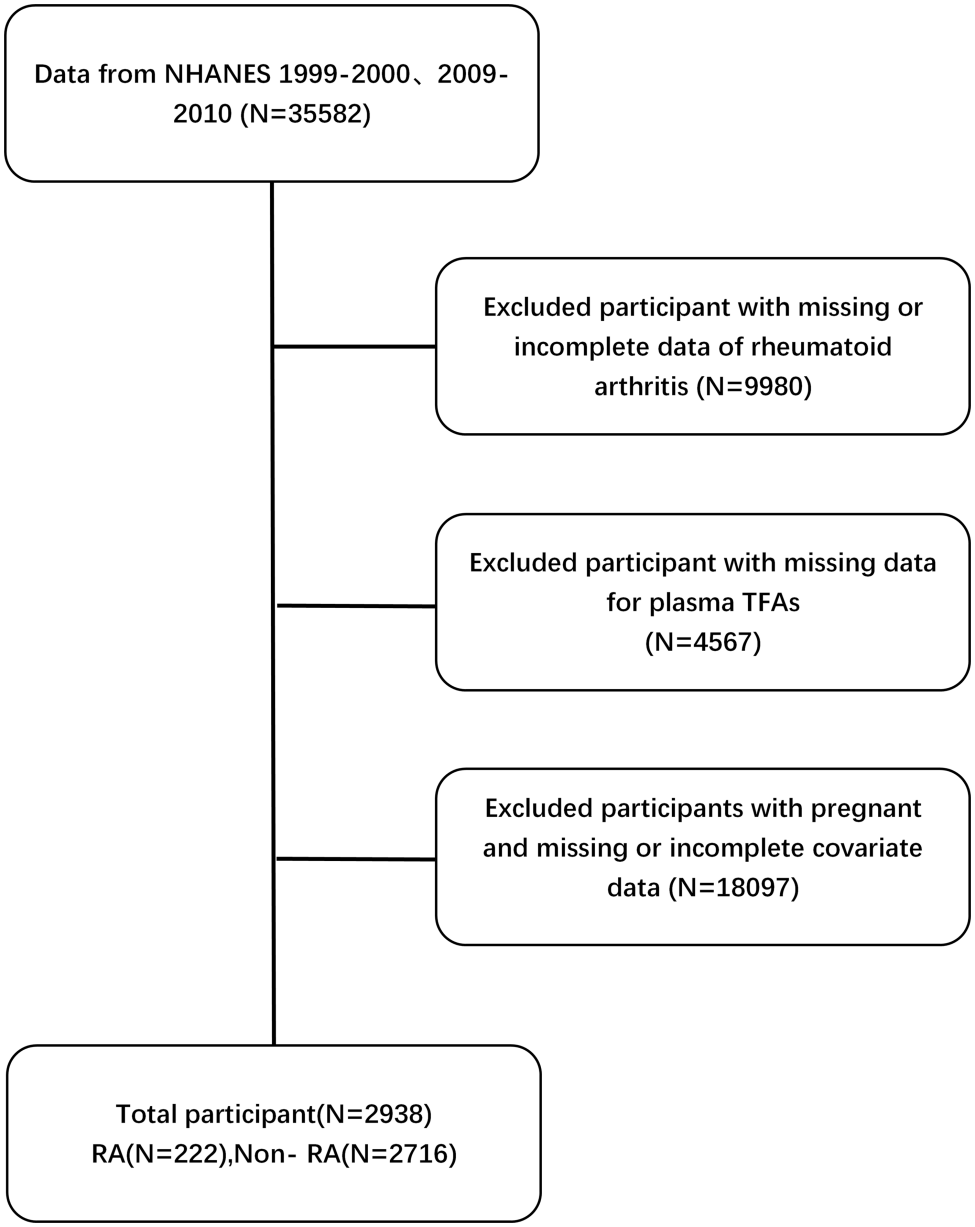
Flowchart of NHANES participant selection in 1999–2000 and 2009–2010.

After applying the exclusion criteria, the study ended up with a final study cohort consisting of 2,938 participants. Please refer to Figure 2 for a more thorough explanation of the sample procedure, exclusion criteria, and research design. This figure provides more detailed information on these aspects.

### Baseline details on the participants

A comparison of the features of participants with and without RA is shown in Table 1. The cohort, which included 2,938 people who satisfied the requirements for enrollment, had a 7.56% RA prevalence. Among the participants, 54.29% were under the age of 50, 48.26% were male, and 63.10% were either married or in a relationship. Additionally, 48.91% had completed high school or more, 32.16% had hypertension, 54.42% had never smoked, and 8.54% had diabetes. In terms of ethnicity, 31.58% identified as Mexican American, 49.97% as non-Hispanic white, 16.41% as non-Hispanic black, and 12.05% as belonging to other ethnicities.

**Table 1:** Characteristics of participants in the 1999–2000 and 2009–2010 NHANES, by RA status.

Variables	Level	Overall	Non-RA	RA	*p*-value
*n*		2,938	2,716	222	
Gender (%)	Male	1,418 (48.26)	1,330 (48.97)	88 (39.64)	0.0075
	Female	1,520 (51.74)	1,386 (51.03)	134 (60.36)	
Age [year; median (IQR)]		47 (33, 63)	46(32, 62)	63 (54, 72)	<0.0001
Age group (%)	≤50	1,595 (54.29)	1,548 (57.00)	47 (21.17)	<0.0001
	>50	1,343 (45.71)	1,168 (43.00)	175 (78.83)	
Ethnicity (%)	Mexican American	634 (21.58)	590 (21.72)	44 (19.82)	0.0042
	Non-Hispanic black	482 (16.41)	429 (15.80)	53 (23.87)	
	Non-Hispanic white	1,468 (49.97)	1,359 (50.04)	109 (49.10)	
	Other	354 (12.05)	338 (12.44)	16 (7.21)	
Education (%)	Below high school	840 (28.59)	744 (27.39)	96 (43.24)	<0.0001
	Completed high school	661 (22.50)	612 (22.53)	49 (22.07)	
	Above high school	1,437 (48.91)	1,360 (50.07)	77 (34.68)	
PIR (%)	<1.3	890 (30.29)	809(29.79)	81 (36.49)	0.083
	1.3–3.5	1,123 (38.22)	1,041 (38.33)	82 (36.94)	
	>3.5	925 (31.48)	866 (31.89)	59 (26.58)	
Marital status (%)	Never married	467 (15.90)	457 (16.83)	10 (4.50)	<0.0001
	Widowed/Divorced/Separated	617 (21.00)	540 (19.88)	77 (34.68)	
	Married/Live with partner	1854 (63.10)	1719 (63.29)	135 (60.81)	
Diabetes (%)	No	2,687 (91.46)	2,513 (92.53)	174 (78.38)	<0.0001
	Yes	251 (8.54)	203 (7.47)	48 (21.62)	
BMI (%)	<25	887 (30.19)	826 (30.41)	61 (27.48)	0.0823
	25–30	1,009 (34.34)	942 (34.68)	67 (30.18)	
	>30	1,042 (35.47)	948 (34.90)	94 (42.34)	
Hypertension (%)	No	1993 (67.84)	1890 (69.59)	103 (46.40)	<0.0001
	Yes	945 (32.16)	826 (30.41)	119 (53.60)	
Work activity (%)	No	1,551 (52.79)	1,419 (52.25)	132 (59.46)	0.0386
	Yes	1,387 (47.21)	1,297 (47.75)	90 (40.54)	
Smoking status (%)	Never	1,599 (54.42)	1,508 (55.52)	91 (40.99)	<0.0001
	Former	740 (25.19)	658 (24.23)	82 (36.94)	
	Now	599 (20.39)	550 (20.25)	49 (22.07)	
Alcohol consumption (%)	No	855 (29.10)	768 (28.28)	87 (39.19)	0.0006
	Yes	2083 (70.90)	1948 (71.72)	135 (60.81)	
Palmitelaidic acid [μmol/L; mean (SD)]		2.281 (0.763)	2.271 (0.765)	2.394 (0.735)	0.0169
Vaccenic acid [μmol/L; mean (SD)]		4.597 (0.883)	4.587 (0.889)	4.721 (0.806)	0.0179
Linolelaidic acid [μmol/L; mean (SD)]		0.974 (0.753)	0.962 (0.754)	1.130 (0.720)	0.0008
Elaidic acid [μmol/L; mean (SD)]		4.286 (0.962)	4.266 (0.964)	4.524 (0.914)	0.0001

### Associations between TFAs and RA

The results of a multivariate logistic regression study that looked at the relationship between RA and four different types of TFAs are shown in Table 2. Only elaidic acid and linolelaidic acid had a significant positive connection with RA in the unadjusted model (1.33, 1.09 ~ 1.62; *p* = 0.006) and (1.52, 1.16 ~ 1.98; *p* = 0.003). The positive connection between linolelaidic acid and RA persisted in Model 2 after controlling for gender, age, ethnicity, education, PIR, and marriage (1.41, 1.10 ~ 1.81; *p* = 0.009). Linolelaidic acid continues to have a substantial and positive correlation with RA (1.39, 1.05 ~ 1.85; *p* = 0.025) even after controlling for all factors in Model 3. This means the chance of getting RA rises by 39% for every unit increase in linolelaidic acid.

**Table 2:** Odd ratios (ORs) and 95% confidence interval (CI) for RA according to TFAs.

TFAs	Model 1		Model 2		Model 3	
	OR (95%CI)	*p-*value	OR (95%CI)	*p-*value	OR (95%CI)	*p-*value
Palmitelaidic acid	1.27 (0.96 ~ 1.67)	0.092	1.15 (0.86 ~ 1.54)	0.33	1.19 (0.85 ~ 1.67)	0.283
Vaccenic acid	1.15 (0.92 ~ 1.45)	0.202	1.1 (0.89 ~ 1.37)	0.35	1.12 (0.88 ~ 1.44)	0.325
Elaidic acid	1.33 (1.09 ~ 1.62)	0.006	1.2 (0.99 ~ 1.45)	0.067	1.19 (0.95 ~ 1.50)	0.12
Linolelaidic acid	1.52 (1.16 ~ 1.98)	0.003	1.41 (1.10 ~ 1.81)	0.009	1.39 (1.05 ~ 1.85)	0.025

OR: odds ratio; 95% Cl: 95% confidence interval. Model 1: No covariates were adjusted. Model 2: Adjusted for gender, age, ethnicity, education level, and PIR. Model 3: Adjusted for gender, age, ethnicity, education level, PIR, marital status, BMI, hypertension, diabetes, work activity, smoking status, and alcohol consumption.

For the sensitivity analysis, this study converted the continuous variable, linolelaidic acid, into categorical variables (quartiles; Table 3). The odds ratios (ORs) and related confidence intervals (CIs), properly adjusted for any confounding variables, showed that the highest quartile Q4 had a 157% higher risk of RA than the lowest quartile Q1(2.57, 1.35–4.88; *p* = 0.01; *p* for trend = 0.012).

**Table 3:** OR and 95%CI for RA according to linolelaidic acid quartile.

	Model 1	Model 2	Model 3
	OR (95%CI) *p*-value	OR (95%CI) *p*-value	OR (95%CI) *p*-value
Continuous			
Linolelaidic acid	1.52 (1.16 ~ 1.98) 0.003	1.41 (1.10 ~ 1.81) 0.009	1.39 (1.05 ~ 1.85) 0.025
Categories			
Q1	Reference	Reference	Reference
Q2	2.09 (1.04 ~ 4.18) 0.038	1.83 (0.90 ~ 3.73) 0.091	1.95 (0.90 ~ 4.23) 0.08
Q3	2.42 (1.40 ~ 4.19) 0.003	2.04 (1.19 ~ 3.50) 0.013	2.04 (1.14 ~ 3.68) 0.023
Q4	3.11 (1.73 ~ 5.58) <0.001	2.56 (1.45 ~ 4.54) 0.003	2.57 (1.35 ~ 4.88) 0.01
*p* for trend	<0.001	0.005	0.012

Linolelaidic acid was converted from a continuous variable to a categorical variable (quartile). OR: odds ratio; 95%Cl: 95% confidence interval. Model 1: No covariates were adjusted. Model 2: Adjusted for gender, age, ethnicity, education level, and PIR. Model 3: Adjusted for gender, age, ethnicity, education level, PIR, marital status, BMI, hypertension, diabetes, work activity, smoking status, and alcohol consumption.

### Linear relationship between RA and linolelaidic acid

**Figure 3:**
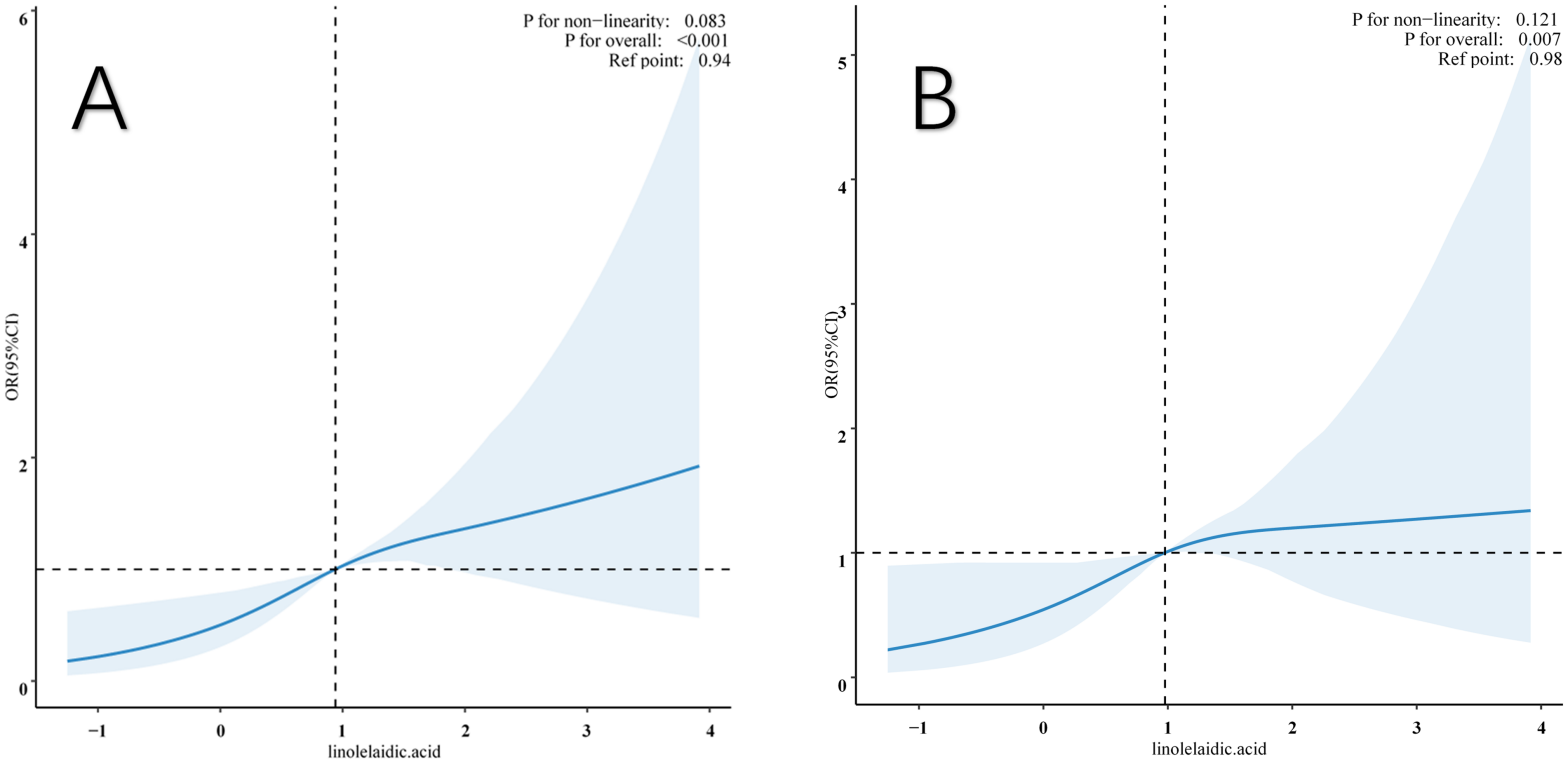
Restricted cubic spline (RCS) illustrates the relationship between linolelaidic acid and RA. The non-adjutsed relationship between linolelaidic acid and RA **(A)**. The full-adjutsed relationship between linolelaidic acid and RA **(B)**.

This study also examined the association between linolelaidic acid and RA risk using a limited cubic spline curve (Figure 3). Linolelaidic acid was discovered to have a linear connection with the risk of RA (Figure 3A), and this relationship remains consistent after adjusting for multiple covariates (as shown in Figure 3B).

Additionally, the study found a threshold impact in the data, with linolelaidic acid having a crucial value of 0.98. When it is below this critical value, the risk of RA increases dramatically, and when it exceeds the critical value, the risk plateaus off. The original model’s *p*-value overall was <0.001, but after all multiple factors were fully adjusted, it was 0.007.

### The subgroup analysis and interaction test

Table 4 displays the results of subgroup analyses that were conducted to ascertain the connection between linolelaidic acid and RA. Notably, several factors, including gender, age, BMI, ethnicity, work activity, alcohol consumption, smoking status, and diabetes showed no significant associations (*p*-value >0.05). However, no significant interactions were observed between the subgroups (*p* for interaction >0.05). Subgroup analysis, stratified by PIR, BMI, and hypertension, revealed some noteworthy findings. The positive correlation between linolelaidic acid and RA was found in PIR>3.5 subjects (1.27, 1.02 ~ 1.59; *p* = 0.03), BMI = 25–30 subjects (1.29, 1.08 ~ 1.55; *p* = 0.01), and hypertensive subjects (1.32, 1.03 ~ 1.68; *p* = 0.03).

**Table 4:** Subgroup analysis for the association between linolelaidic acid and RA, weighted.

Subgroup	OR(95%CI)	*p-*value	*p* for interaction
Gender			0.615
Male	1.08 (0.94 ~ 1.24)	0.26	
Female	1.19 (0.99 ~ 1.42)	0.06	
Age group			0.411
≤50	1.06 (0.84 ~ 1.33)	0.61	
>50	1.17 (1.00 ~ 1.38)	0.05	
Ethnicity			0.438
Mexican American	0.76 (0.46 ~ 1.26)	0.18	
Non-Hispanic black	1.14 (0.75 ~ 1.75)	0.44	
Non-Hispanic white	1.13 (0.97 ~ 1.31)	0.1	
Other	1.84 (0.66 ~ 5.12)	0.16	
PIR			0.173
<1.3	1.17 (0.92 ~ 1.49)	0.19	
1.3–3.5	0.94 (0.72 ~ 1.24)	0.65	
>3.5	1.27 (1.02 ~ 1.59)	0.03	
BMI			0.284
<25	0.89 (0.68 ~ 1.17)	0.37	
25–30	1.29 (1.08 ~ 1.55)	0.01	
>30	1.12 (0.89 ~ 1.40)	0.3	
Work activity			0.956
No	1.12 (0.97 ~ 1.30)	0.1	
Yes	1.14(0.90 ~ 1.44)	0.25	
Alcohol consumption			0.691
No	1.21 (0.90 ~ 1.61)	0.18	
Yes	1.09 (0.95 ~ 1.26)	0.18	
Smoking status			0.503
Never	1.18 (0.98 ~ 1.41)	0.07	
Former	1.19 (0.94 ~ 1.50)	0.13	
Now	0.85 (0.61 ~ 1.20)	0.32	
Hypertension			0.126
No	1.02 (0.89 ~ 1.17)	0.72	
Yes	1.32 (1.03 ~ 1.68)	0.03	
Diabetes			0.383
No	1.14 (1.00 ~ 1.31)	0.06	
Yes	0.87 (0.49 ~ 1.56)	0.51	

## Discussion

The analysis has revealed a correlation between plasma linolelaidic acid and RA. In a fully adjusted model, this connection remained after controlling for pertinent confounding factors. Subgroup analyses and interaction tests have suggested that plasma linolelaidic acid may play a crucial role in identifying RA in patients who have a PIR greater than 3.5, are BMI = 25–30, and have hypertension. A linear association with a breakpoint of 0.98 has been found between linolelaidic acid and RA, according to regression curve fitting and cutoff effect analyses. The study highlighted the potential role of trans fats in determining the likelihood of developing RA.

The exact mechanisms that connect plasma TFAs and RA are still unclear. However, various observational and experimental studies suggest that trans fats have pro-inflammatory effects. Consumption of trans fats can increase levels of inflammatory markers in the blood, such as CCL2, CRP, IL-6, IL-1β, and TNF-α (11, 23, 24).

RA is an inflammatory syndrome that prompts synovial joints to become chronically inflamed (25). The expansion of synovial cells and the invasion of activated immune-inflammatory cells, including memory T lymphocytes, phagocytes, and plasma cells, are the causes of this inflammation, which eventually destroys cartilage as well as bone structure (26). The inflammatory process is believed to continue because of cytokines, such as IL-6, IL-1β, and TNF-α, among others (27). In addition, joint swelling in rheumatoid arthritis is caused by an immune response that leads to inflammation in the synovial chamber. This inflammation is regulated by a complex network of cytokines and chemokines. Clinical interventions have shown that tumor necrosis factor (TNF), interleukin 6, and possibly granulocyte–monocyte colony-stimulating factors are critical components of this process (28). Cytokines and chemokines activate endothelial cells and attract immune cells to accumulate in the synovial chamber, triggering or worsening inflammatory responses (29). Therefore, elevated inflammatory markers in the blood and an increase in immune-inflammatory cytokines may be one of the ways that trans fatty acids are linked to rheumatoid arthritis.

Excessive consumption of TFAs can be harmful to cells as they regulate the production of reactive oxygen species (ROS) and cause oxidative stress (30). Studies in mice have shown that a high intake of trans fatty acids leads to inflammation and oxidative stress (31). Research on Wistar rats has demonstrated that the presence of industrial trans fatty acids in their diet increases oxidative stress and the levels of catalase and superoxide dismutase, while decreasing the activity of antioxidant enzymes. This results in an increase in hepatic lipotoxicity (32). ROS production and oxidative stress are also mechanisms behind rheumatoid arthritis. Previous studies have established a positive correlation between ROS and RA severity (33–35). Changes in ROS levels can lead to oxidative stress in RA patients (36), resulting in biomolecular and tissue damage (37), stimulating the inflammatory response of living systems (38), and causing chronic diseases. As such, increased ROS production and oxidative stress may explain the relationship between trans fatty acids and rheumatoid disease.

Previous research has revealed that industrially produced trans fatty acids (iTFAs) are more detrimental to health than naturally occurring trans fatty acids (nTFAs) found in ruminants (39). Although linolelaidic acid, which is mainly derived from industrial fatty acids, is harmful to health, it is generally recommended to reduce the intake of all types of TFAs because it is difficult to determine whether the trans fats in the food are from industrial or natural sources. Regarding the intake of TFAs, the Food and Agriculture Organization of the United Nations (FAO) and the World Health Organization (WHO) have proposed that the daily intake should be lower than 1% of the total dietary calories (40). Nevertheless, certain previous studies have revealed that approximately 90% of adult individuals in the United States consume foods containing TFAs. Approximately 16% of American adults have ingested more than 0.5% of their total energy from TFAs within the past 24 h, among which 8% of American adults have their TFA intake accounting for more than 1% of the total energy (41). A systematic review targeting the intake of trans fatty acids among the general population in 29 countries worldwide has indicated that although the intake of industrial trans fatty acids has significantly declined since 1995, the intake level of trans fatty acids in seven countries still exceeds the 1% recommended by the WHO (6). In 2018, the WHO initiated the “REPLACE” action plan, to eliminate trans fats from the global food industry by 2023. Following the announcement of REPLACE, multiple countries have adhered to strict regulations and guidelines to restrict the supply of trans fats in various types of foods (42). Our research objective is highly in line with this health policy.

### Research advantages and limitations

This is the first descriptive research project delving into the connection between RA and TFAs. The study has the following advantages. First, it provides evidence of a harmful association between linolelaidic acid and RA. Second, TFAs can only be obtained from the daily diet and cannot be synthesized by the body. Therefore, the subtypes of TFAs in the plasma can effectively avoid the recall bias of dietary questionnaires. Additionally, this study used data from a nationally representative multi-ethnic survey in the United States. A weighted logistic regression model was used for analysis, and other covariates were adjusted. These measures enhance the accuracy, reliability, and generalizability of the study’s conclusions to a wider population.

Nonetheless, it is critical to recognize this study’s imperfections. First, the NHANES dataset used was cross-sectional and lacked longitudinal follow-up data. Although the study identified a negative association between plasma linolelaidic acid and RA, there may be a reverse causality in which RA may influence dietary TFA intake through dietary changes. Second, even when several significant confounders were eliminated, residual, or unmeasured confounders may still exist. Third, this study only examined four representative plasma TFA subtypes, and the possible effects of other subtypes remain unclear. Additionally, it is important to note that the arthritis diagnosis in this study was based on patient self-reporting, which may be subject to measurement errors and recall biases. Furthermore, the limitations of the NHANES data prevented us from determining whether participants had other autoimmune or inflammatory diseases in addition to their TFA intake. Additionally, we were unable to obtain key inflammatory factors, such as TNF-α, interleukin-6, and interleukin-10. We hope this study serves as a reference for future research exploring the relationship between TFAs and RA by including a broader range of diseases and inflammatory factors.

## Conclusion

This nationally representative study found a significant association between linolelaidic acid and the development of RA in adults in the United States, even at relatively low levels of exposure. Vaccenic acid, palmitelaidic acid, and elaidic acid were positively correlated, but not significantly so. Therefore, these findings provide evidence supporting the need to reduce dietary intake of trans fatty acids in the general population. Additionally, the study suggests increased awareness of TFA intake in patients with RA, which may provide new insights into the prevention of RA from a dietary perspective.

## Data Availability

The original contributions presented in the study are included in the article/Supplementary material, further inquiries can be directed to the corresponding author.
